# Gold Nanoparticles Functionalized with Angiogenin for Wound Care Application

**DOI:** 10.3390/nano11010201

**Published:** 2021-01-14

**Authors:** Lorena Maria Cucci, Giuseppe Trapani, Örjan Hansson, Diego La Mendola, Cristina Satriano

**Affiliations:** 1 Laboratory of Hybrid NanoBioInterfaces (NHBIL), Department of Chemical Sciences, University of Catania, 95125 Catania, Italy; lorena.cucci@unict.it; 2 Scuola Superiore di Catania, University of Catania, 95123 Catania, Italy; giuseppe.trapani@studium.unict.it; 3 Department of Chemistry and Molecular Biology, University of Gothenburg, SE-40530 Göteborg, Sweden; orjan.hansson@chem.gu.se; 4 Department of Pharmacy, University of Pisa, 56126 Pisa, Italy

**Keywords:** angiogenesis, nanomaterial, plasmonics, nanomedicine, copper, endothelial cells, confocal microscopy, QCM-D, AFM, mutant protein

## Abstract

In this work, we aimed to develop a hybrid theranostic nano-formulation based on gold nanoparticles (AuNP)—having a known anti-angiogenic character—and the angiogenin (ANG), in order to tune the angiogenesis-related phases involved in the multifaceted process of the wound healing. To this purpose, spherical were surface “decorated” with three variants of the protein, namely, the recombinant (rANG), the wild-type, physiologically present in the human plasma (wtANG) and a new mutant with a cysteine substitution of the serine at the residue 28 (S28CANG). The hybrid biointerface between AuNP and ANG was scrutinized by a multi-technique approach based on dynamic light scattering, spectroscopic (UV-visible, circular dichroism) and microscopic (atomic force and laser scanning confocal) techniques. The analyses of optical features of plasmonic gold nanoparticles allowed for discrimination of different adsorption modes—i.e.; predominant physisorption and/or chemisorption—triggered by the ANG primary sequence. Biophysical experiments with supported lipid bilayers (SLB), an artificial model of cell membrane, were performed by means of quartz crystal microbalance with dissipation monitoring acoustic sensing technique. Cellular experiments on human umbilical vein endothelial cells (HUVEC), in the absence or presence of copper—another co-player of angiogenesis—were carried out to assay the nanotoxicity of the hybrid protein-gold nanoassemblies as well as their effect on cell migration and tubulogenesis. Results pointed to the promising potential of these nanoplatforms, especially the new hybrid Au-S28CANG obtained with the covalent grafting of the mutant on the gold surface, for the modulation of angiogenesis processes in wound care.

## 1. Introduction

Cutaneous wound healing is an essential physiological process consisting of a sequence of molecular and cellular events, which occur to restore lesions induced by specific trauma or pathological conditions that break the physical continuity of functional tissues [1].

Impaired wound healing can result in severe damage to the deep layers of the skin and could lead to high medical burden and poor quality of life. Several wound treatments have been proposed to speed up the healing process, thus reducing infections and scar formations like cellular bandages [2], light treatments [3], electrical stimulation [4] and pharmacotherapy [5].

Cellular and biochemical events in wound repair can be divided into four stages: bleeding and homeostasis, inflammatory reaction, cell proliferation and remodeling [6]. In the latter stages of wound healing new capillaries invade the wound space, thus allowing the new tissue to receive sufficient oxygen and nutrients [7]. This process involves several cell types, including fibroblasts, macrophages and endothelial cells, as well as the action of many soluble molecules capable to regulate cells interactions and to stimulate wound angiogenesis. Among them, angiogenin (ANG), which is the most abundant factor in the wound environment, plays a pivotal role. In particular, it has been found that the high level of ANG in the wound fluids are able to induce endothelial cells proliferation and circular angiogenic cell differentiation, while the antibody neutralization of ANG in equivalent wound fluids leads to a reduction of their angiogenic properties [8].

Literature data reported high levels of angiogenin in human burn wounds and deep thickness lesions, thus supporting a pivotal role of ANG in the wound management [9].

Copper, an essential mineral for humans, plays also a crucial role in the process of angiogenesis, as well as in all the stages of the wound healing management. In the epithelial tissue, indeed, copper stimulates dermal fibroblast proliferation [10], upregulates collagen, promotes elastin fiber components formation (fibrillins) [11], stabilizes the extracellular matrix (ECM) protein cross-linking [12] and as a cofactor of the superoxide dismutase, inhibits cellular oxidative effects due to the action of free radicals [13]. In this regard, it has been demonstrated that copper ions are able to stimulate endothelial cell migration and neovascularization in avascular rabbit corneas while its depletion, by Cu-chelators, such as penicillamine and trientine, in vivo, prevents the vessels formation [14,15]. Such an intersection between copper and angiogenesis supports the critical role of the metal in pathological and physiological angiogenic processes, such as wound repair where a higher concentration of copper ions (~30 μM) has been detected with respect to the peri-wound areas [16,17]. Hence, the local modulation of copper pro-angiogenic effect provides a promising strategy to enhance tissue repair and regeneration.

The mechanism behind the pro-angiogenic activity of copper is the activation and amplification of the angiogenic response triggered by several cytokines and proteins including hypoxia inducible factor-1 (HIF-1), vascular endothelial growth factor (VEGF), fibroblast growth factor (FGF) and ANG, through the formation of metal complexes, leading to conformational changes and modulating their biological activity [18]. During the early stages of angiogenesis, the intracellular copper has been demonstrated to stabilize HIF-1 structure, thereby promoting its transcriptional activity on angiogenic genes including VEGF and ceruloplasmin genes [19] while, experiments on cultured human cardiomyocytes showed that copper ions, at the concentration of 5 μM, stimulate insulin-like growth factor-1 (IGF-1)-induced VEGF expression [20]. Furthermore, copper complexes of the fibroblast growth factor 1 (FGF-1), stimulate FGF secretion and ECM degradation, thus promoting cell migration and proliferation [21].

Recently, hybrid nanoplatforms, where the biomolecule of interest is immobilized in a suitable way at the surface of a given material, have attracted a lot of interest in order to get synergic and/or enhanced response at the biointerface [22,23,24,25]. In particular, in previous works, we have shown that gold nanoparticles functionalized at their surface with angiogenin-mimicking peptides and/or the whole protein exhibited a tuneable interaction with the cell membrane [26], thus providing a promising therapeutic approach in anti-cancer therapy of brain tumors [27,28].

The biggest advantage of nanomaterials in tissue repair, is the high surface to volume ratio (S/V) and the small diameter (<100 nm) [29], which match with the natural tissue fibers of the skin while, their mechanical properties, such as light stiffness, porosity and low molecular weight, prevent the compression of the injured tissue in situ [30].

In the wide class of nanomaterials, metal nanoparticles and in particular gold nanoparticles, are extensively used for biological applications, due to their size-related electronic [31], magnetic [32] and optical properties [33], as well as their straightforward functionalization.

As to the optical properties, gold nanoparticles (AuNP)s show a strong absorption band in the visible range, due to the interaction of the light with the unbound valence electrons of the metal. This phenomenon is termed surface plasmon resonance (SPR) and allows for the application of the nanoparticles as biosensors and theranostic systems [34,35,36]. Noteworthy, the change in the optical plasmonic features of metal nanoparticles upon the immobilization of a biomolecule at their surface can be very useful to discriminate among different biomolecule conformations at the interface with the metal [37,38,39].

Physical and chemical interactions can be used for the decoration with biomolecules (such as proteins, antibodies, lipids, DNA) and/or drugs of gold nanoparticles [40]. In the case of gold nanoparticles, physical interactions may depend on three concurrent effects: (i) ionic attraction between the negatively charged citrate capped-AuNPs and the positively charged groups of the molecules; (ii) hydrophobic interactions; (iii) dative binding between the gold conducting electrons and amino acid sulfur atoms. On the other hand, chemical interactions can take place through: (i) Au-S bonds with thiol groups; (ii) bifunctional linkers and (iii) adapter molecules like streptavidin and biotin [41,42,43].

During the healing process, the immune system produces a large amount of reactive oxygen species (ROS), which inhibit cell growth and cause damages to DNA, RNA and proteins, thus impairing and slowing down the physiological wound repair [44]. AuNPs are able to quench the action of ROS like hydroxyl (OH), hydrogen peroxide (H_2_O_2_) and nitric oxide (NO), hence may show also anti-oxidative effects [45]. Their large surface area, indeed, allows the nanostructures to easily accept electrons from ROS, leading to their deactivation [46,47,48]. In this perspective, AuNPs, may play a central role in the wound care treatment [49,50].

In the present work, with the aim to set up a nanomedicine platform able to tune the angiogenesis processes during wound healing, we scrutinized a multifaceted hybrid system based on AuNP, ANG and copper. In particular, we studied three variants of ANG: (i) the recombinant protein, rANG; (ii) the wild-type angiogenin, wtANG; (iii) a new mutant, S28CANG, with a cysteine instead of the serine at the residue 28, for the high affinity binding to the metal surface, through Au-S bonds. To note, the serine 28 is an ideal residue for the cysteine substitution, since it is placed on the surface of the protein and out of its active site pocket, thus the single point mutation at the serine permits to maintain the biological activity of ANG as well as allows for a predominant covalent grafting of the protein onto the surface of the metal nanoparticle. Moreover, serine and cysteine present a similar chemical structure and a comparable side-chain volume, since they differ only at the γ atom, namely oxygen for the serine and sulfur for the cysteine, respectively. Accordingly, a predominant chemisorption mechanism is expected at the interface with AuNPs for the mutant protein S28CAng, while a “pure” physisorption process would occur for both wtANG and rANG, respectively.

The hybrid AuNP/ANG systems were assembled and characterized by physicochemical and biophysical approaches to scrutinize, in the absence or presence of copper ions: (i) their interaction with artificial cell membranes made of supported lipid bilayers (SLBs), by means of the acoustic sensing technique, Quartz Crystal Microbalance with Dissipation (QCM-D) monitoring; (ii) their effect on HUVEC, a common cell model to study the wound healing process, in terms of cytotoxicity (MTT test), cell migration (scratching test) and tubulogenesis (Matrigel test).

Also, the effect of the studied systems on the monovalent copper intracellular distribution was investigated by laser scanning confocal microscopy (LSM), by using the specific BODIPY-Cu(I) fluorescent sensor (CS1) [51]. The conformational features of the three variants of ANG and the structural changes induced by the copper (II) complex formation were investigated by Circular Dichroism (CD) spectroscopy. The surface functionalization of the gold nanoparticles, for both physisorption and chemisorption, with distinctive arrangement of the three proteins at the interface with AuNPs, was scrutinized by means of UV-visible spectroscopy, by inspecting the plasmonic changes. Atomic force microscopy (AFM) and dynamic light scattering (DLS) analyses were carried out to assess the morphological features/coverage and hydrodynamic size of hybrid AuNP/ANG systems, respectively.

## 2. Materials and Methods

### 2.1. Chemicals

Gold(III) chloride trihydrate and trisodium citrate dihydrate were purchased from Sigma-Aldrich (St. Louis, MO, USA). Ultrapure MilliQ water was used (18.2 mΩ·cm at 25 °C, Millipore, (Darmstadt, Germany). 3-(N-morpholino)-propane sulfonic acid (MOPS) buffer solution (added with 0.003 M KCl and 0.14 M NaCl) was prepared at the concentration of 1 mM and pH was corrected to 7.4. Glassware was cleaned with aqua-regia rinsing (HCl:HNO_3_, 1:3 volume ratio) and then washed with MilliQ water before starting the experiments. Small unilamellar vesicles (SUVs) were prepared from 1-palmitoyl-2-oleoylsn-glycero-3-phosphocholine (POPC) and the NBD-labelled, 1,2-dipalmitoyl-sn-glycero-3-phosphoethanolamine-N-(7-nitro-2-1,3-benzoxadiazol-4-yl) (ammonium salt) (PE-NBD), purchased from Avanti Polar Lipids (Alabaster, AL, USA). Phosphate buffer saline (PBS) solution (0.01 M phosphate buffer, containing 0.003 M KCl and 0.14 M NaCl, pH 7.4) was prepared from tablets (Sigma-Aldrich, St. Louis, MO, USA). The designed primers for Ang protein expression were purchased from Eurofins GWM (Ebersberg, Germany). The over-expression plasmid (pET22b(+)-ANG), including a codon-optimized gene for ANG, was obtained from Sloning BioTechnology (Puchheim, Germany). For the protein expression, yeast extract and glycerol, were purchased from Merck, bacto-peptone from DIFCO, tris(hydroxymethyl)aminomethane (TRIS) from AMRESCO and isopropyl β-D-1-thiogalactopyranoside (IPTG) from VWR, while the other reagents were obtained from Sigma Aldrich (St. Louis, MO, USA) at the highest commercially available grade. Medium200 supplemented with low serum growth factor (LSGS) was purchased from Gibco. Penicillin/Streptomycin, Amphotericin B solutions and gelatine were provided by Sigma Aldrich (St. Louis, MO, USA).

### 2.2. Expression and Purification of Recombinant Angiogenin (rANG)

The human ANG expression was carried out following the method reported by Holloway et al. [52]. Briefly, the *E. Coli* (BL21(DE3)) expression strain was cultured at 37 °C under shaking (180 rpm) in 5 mL of terrific broth (12 g peptone, 24 g granulated yeast extract, 4 mL glycerol 87%, 900 mL of MilliQ water) supplemented with ampicillin (100 μg/mL). After 24 h of incubation the whole volume of the bacterial culture was inoculated in 1000 mL of fresh broth. When the density of the culture had reached the OD_600nm_ value of 0.8, the ANG expression was induced by the addition of 1 mM isopropyl β-D-1-thiogalactopyranoside (IPTG) and the incubation was continued for additional 2 h. Afterwards, the cell culture was harvested by centrifugation (4000 RCF for 15 min at 4°C, JLA 8,100) and cells were lysed with 30 mL of lysis buffer (50 mM Tris-HCl, 2 mM EDTA, pH = 8) by using the high-pressure homogenizer (Emulsiflex) and a sonication step (Qsonica Sonicator Q700, Newtown, CT, USA). Lysate was, then, centrifuged (20,000 RCF for 40 min at 4° C, JA25.50) and the pellet was re-suspended in 25 mL of lysis buffer supplemented with 1% (*v*/*v*) Triton X-100. Sonication and centrifugation steps were repeated twice, and the final pellet was dissolved in 30 mL of denaturation buffer (0.24 M guanidine hydrochloride (GdnHCl), 100 mM Tris-HCl, 1 mM ethylenediaminetetraacetic acid (EDTA), 4 mM NaCl, 0.4 mM 1,4-dithiothreitol (DTT)).

The expressed rANG was refolded from inclusion bodies according to the procedure described by Jang et al. (2004) [53] and then purified by a cation exchange chromatography performed on an automated chromatographic workstation (Akta prime, GE Healthcare, Chicago, Illinois, USA) equipped with a 15 × 1.6 cm column packed with SP Sepharose Fast Flow (GE Healthcare, Chicago, Illinois, USA). After a washing step with 25 mM Tris-HCl (pH = 8.0), rANG was eluted with 25 mM Tris-HCl, 1 M NaCl (pH = 8.0) buffer solution.

### 2.3. Expression and Purification of Wild Type Angiogenin (wtANG)

The rANG was incubated with 1 nM *Aeromonas* aminopeptidase, at the concentration of 1 × 10^−5^ M in 200 mM potassium phosphate buffer (pH = 7.2) (overnight at 37 °C under gentle shaking). This procedure allows for the specific removal of the N-terminal methionine residue (Met(-1)) in the primary sequence of rANG, thus obtaining the N-terminal glutamine residue (Glu1) that spontaneously cyclizes to the pyroglutamate residue (PyrGlu1) that is characteristic of ”native” wtANG. The reaction mixture was purified by dialysis (Spectra/por MWCO 6–8000 Da), which replaces PBS with 25 mM Tris-HCl (pH 7.4) buffer solution, followed by cation-exchange chromatography.

### 2.4. Site-Directed Mutagenesis of ANG: Expression of the Mutant S28CANG

To introduce the substitution in the ANG gene, the QuickChange Lightning site-directed mutagenesis kit was used. A pair of oligonucleotide primers (forward and reverse), homologous to the template DNA, were accurately designed according to the chosen mutation. Thus, the codon AGT (for Ser) was mutated to TGT (for Cys), and the oligonucleotide primers were constructed as follows:

(Forward) 5′-GAT CGC TAC TGT GAG **TGT** ATC ATG CGT CGT CGC GG-3′

(Reverse) 5′-CC GCG ACG ACG CAT GAT ACA CTC ACA GTA GCG ATC-3′

The designed primers were characterized by a melting temperature ($T_{m}$) of 82.6 °C, as estimated by the following formula (Equation (1)):

(1) $$T_{m}=81.5+0.41\;\left(\%NG\;NC\right)+\left(\frac{675}{N}\right)-\left(\%\;mismatch\right)$$

where N is the number of nucleotides (N=35 in our case) and NG and NC are referred to the number of guanine (NG=12 for the forward and 7 for the reverse) and cytosine (NC=7 for the forward and 12 for the reverse) bases, contained within the sequence, respectively.

Polymerase chain reaction (PCR) was performed to conduct the mutagenesis process that consists of the following three steps: (1) the denaturation by heating of the template DNA molecules; (2) the annealing of the primers to the parental strands; (3) the amplification of the mutated plasmid by DNA polymerase. PCR was carried out according to the manufacturer’s instructions. Briefly, 5 μL of QuickChange buffer, 1 μL of deoxynucleotides (dNTPs), 100 ng of plasmid pET22b(+)-ANG, 125 ng of each primer, 1.5 μL of QuickSolution reagent and nuclease free-H_2_O to reach a final volume of 50 μL, were sequentially added to the PCR-tube to prepare the sample reaction. Finally, 1 μL of PfuUltra high-fidelity DNA polymerase was rapidly added to the reaction mixture and the sample was thermo-cycled following the steps showed in the table below (Table 1).

To test the efficiency of the DNA mutation, a control reaction was similarly prepared by adding 25 ng of the plasmid pWhitescript 4.5-kb to the reaction mixture.

After the completion of the PCR cycling, the obtained products were treated with 2 μL of Dpn I restriction enzyme, which specifically digests the 5’-Gm6ATC-3´ sequence in the parental template DNA. The uncut mutated DNA sequences were used to transform the XL10-Gold ultra-competent cells. Thus, 2 μL of Dpn I-treated DNA from each control and sample reaction were transferred to pre-chilled tubes containing 45 μL of cells and 2 μL of β- mercaptoethanol (ME) mix provided with the kit. The cell suspensions were incubated on ice for 30 min, heat-shocked in a 42 °C water bath for 30 s and finally placed on ice for 2 min. 250 μL of NZY+ broth (10 g/L NZ amine, 5 g/L NaCl, 5 g/L Yeast extract, 12.5 mM MgCl_2_, 12.5 mM MgSO_4_, 40 g/L glucose) were, subsequently, added to each tube and the cellular suspensions were incubated at 37 °C for 1 h with shaking at 225 rpm.

Afterwards, 200 μL of each transformation reaction were plated on Luria-Bertani (LB) agar plates supplemented with ampicillin, as the appropriate antibiotic for the used plasmid vector and then incubated at 37 °C overnight. After 24 h of incubation, a high presence of bacterial colonies was observed on all the tested plates and as expected no colonies of no transformed XL10-Gold ultra-competent cells grew in presence of ampicillin, thus verifying the effective cells transformation with the control and mutated DNA.

Individual colonies were transferred to 5 mL of LB broth supplemented with 0.1 mg/mL of ampicillin and subsequently incubated overnight, at 37 °C under shaking at 180 rpm. Afterwards, plasmid DNA purification was performed through the Gene Jet Plasmid Miniprep Kit (Thermo-Scientific, Waltham, MA, USA) according to manufacturer’s instructions and analyzed by DNA sequencing (Eurofins MWG Operon, Ebersberg bei München, Germany). Finally, BL21 cells, specifically studied to have a high-level expression of recombinant proteins, were transformed with the mutated plasmid by heat-shock method using the One Shot^®^ BL21(DE3) Chemically Competent *E. coli* cells. The transformed bacterial culture was plated on LB + ampicillin agar plates for 24 h at 37 °C and the obtained colonies were transferred to grow into 5 mL of LB medium, supplemented with 0.1 mg/mL of ampicillin. Cells were incubated overnight at 37 °C, under shaking at 225 rpm and finally frozen after the addition of 10% (*v*/*v*) glycerol.

The S28CANG mutant protein was expressed and purified as described for the native protein in Section 2.3. Noteworthy, to chemically remove the intermolecular S-S bridges formed between the free SH groups of the introduced Cys residue into the amino acid sequence of ANG, without destroying the intramolecular and rather buried disulphide bridges of ANG, the purified S28CANG protein was treated, according to the Levison M.E. et al. procedure, with tris(2-carboxyethyl)phosphine hydrochloride (TCEP) at 5-fold molar excess over the theoretical disulphide bridges concentration, for 20 min at room temperature [54,55].

### 2.5. Circular Dichroism (CD)

CD spectra were recorded on a Jasco model 810 spectropolarimeter, in the 195–270 nm wavelength region, at RT and under a constant flow of nitrogen. Spectra were obtained at the scan rate of 50 nm/min and a resolution of 1 nm using quartz cuvettes with 0.1 cm optical path length as an average of 10 scans. The protein and the gold nanoparticles concentration were 2 × 10^−8^ M and 5 × 10^−9^ M respectively, in 1 mM MOPS buffer at pH 7.4.

### 2.6. Enzymatic Activity

Ribonuclease (RNase) assay was performed to attest the maintenance of the biological activity of the mutant S28CANG protein in comparison with the human wtANG.

The ribonucleolytic activity toward tRNA, was determined by measuring the formation of perchloric acid-soluble fragments, following the procedure reported by Shapiro et al. 1987 [56] and modified by Halloway et al. 2001 [52]. Briefly, wtAng, S28CAng (protein concentrations ranging from 0.5 up to 1.5 μg/mL) and tRNA (20 μg/mL) were incubated for 2 h at 37 °C in 33 mM MOPS buffer solution added with 33 mM NaCl, at the final volume of 300 μL. Afterwards, samples were diluted with 700 μL of ice-cold 3.4% HClO_4_ and kept on ice for an additional 10 min. Finally, samples were centrifuged (17,949 RCF, for 10 min) and the absorbance at 262 nm of perchloric acid tRNA soluble fragments was spectroscopically measured.

### 2.7. Preparation of Gold Nanoparticles (AuNPs)

Gold nanoparticles were synthesized following the method pioneered by J. Turkevich, which uses the chemical reduction reaction of chloroauric acid by the action of trisodium citrate, which acts both as reducing agent and capping agent [57]. Briefly, 20 μL of 1 M gold (III) chloride dihydrate solution were diluted in 20 mL of ultrapure MilliQ water in a 50 mL glass beaker at the final concentration of 1 mM. The solution was brought at the boiling point on a hot plate, under stirring and 2 mL of 1% (*w*/*v*) trisodium citrate dihydrate solution were quickly added to the final concentration of 3.08 mM citrate (Au^3+^/citrate molar ratio of 1:3). As soon as the color turned from yellow to deep red, AuNPs were formed and the beaker was removed from the hot plate. Such a synthetic method produces about 14 nm diameter particles with a plasmonic peak centered at 520 nm [58]. The resultant nanoparticles were centrifuged twice through (8000 rpm, for 15 min), with a washing with 1 mM MOPS buffer (pH = 7.4) in between the two centrifugation steps, in order to remove the excess of sodium citrate from the nanoparticles dispersion and to collect a concentrated pellet. The molar concentration of the AuNP suspension was calculated by using the Lambert Beer low, with the molar extinction coefficient of AuNP calculated as described in the following (according to Navarro et al.; ref. [56]) The average core diameter size (d) of citrate-capped AuNP was estimated by UV-vis spectra, according to Equation (2):

λ_max_ = 515.04 + 0.3647 d, (2)

where λ_max_ is the maximum absorption peak [58]. Hence, the molar concentration of AuNP dispersion was calculated according to Equation (3):

ε = Ad^γ^, (3)

where ε (M^−1^cm^−1^) is the extinction coefficient, d is the core diameter of the nanoparticles previously calculated, and γ = 3.30, A = 4.7 × 10^4^ M^−1^cm^−1^ for d ≤ 85 nm, γ = 1.47, A = 1.6 × 10^8^ M^−1^cm^−1^ for d > 85 nm [59]. In order to calculate the *NP* number per mL, considering the spherical shape of the nanoparticle and a uniform face centered cubic (fcc) structure of the nanoclusters, with the length parameter of unit cell of L = 0.408 nm [38,60], the unit cell volume value (*V_cell_* = L^3^ = 0.0679 nm^3^) was calculated to obtain the volume of a particle, $V_{\mathrm{NP}}$, given by Equation (4):

(4) $$V_{NP}=\;\frac{4}{3}\;\pi \;{\left(\frac{d}{2}\right)}^{3}$$

As well as the mole number of metal atoms for each nanoparticle, $n_{NP}$ [38]:

(5) $$n_{NP}=4\times \frac{V}{V_{cell\cdot }N_{A}}$$

where *N_A_* is the Avogadro’s constant (6.02 × 10^23^ atoms/mol) and 4 is the number of Au atoms that contains one-unit cell. Therefore, the nanoparticle concentration in *NP*/mL is calculated from the molar concentration c and the $n_{NP}$ value as described in Equation (6):

(6) $$NP/\mathrm{mL}=\frac{c}{n_{NP}}$$

### 2.8. Functionalization of AuNPs with the Three Proteins rANG, wtANG, S28CANG

The AuNP pellets, obtained as described in Section 2.6, were resuspended in 1 mM MOPS (pH = 7.4) and then functionalized by predominant physical adsorption (for rANG and wtANG), or chemisorption (for S28CANG), respectively. The concentrations of nanoparticles and proteins used for the immobilization protocol are reported in Table 2.

Such values correspond to the limit concentration of protein added to the nanoparticle dispersion, which ensured the saturation in the shifts of the plasmonic peak (i.e.; the maximum coverage of the nanoparticle) before reaching the aggregation. Eventually, to rinse off unbounded or weakly bound proteins, the functionalized nanosystems were washed by two centrifugation steps (8000 rpm, 15 min) in 1 mM MOPS.

### 2.9. UV–Visible Spectroscopy and Dynamic Light Scattering (DLS) Analysis

UV-vis spectroscopy was performed on the aqueous dispersions of AuNPs, in quartz cuvettes with 1 and 0.1 cm optical path length, in the wavelength range 200–700 nm, at 25 °C, with a spectral resolution of 1 nm on a Perkin Elmer UV-vis spectrometer (Lambda 2S, Waltham, MA, USA). Samples were pre-equilibrated prior to the initial scan for 2 min at RT. Particle size analysis was performed by dynamic light scattering (Horiba LB-550, Kyoto, Japan) and the results were presented as the mean of at least three measurements.

### 2.10. Atomic Force Microscopy (AFM)

To perform AFM imaging, a drop of the sample was put on freshly cleaved muscovite mica (Ted Pella, Inc.; Redding, CA, USA) and incubated at room temperature for 5 min. After that, samples were washed with 1 mL of ultrapure water, dried under a gentle nitrogen stream and immediately imaged. A Cypher AFM instrument (Asylum Research, Oxford Instruments, Santa Barbara, CA, USA) operating in tapping AC-mode was used. Tetrahedral tips made of silicon and mounted on rectangular 30-μm long cantilevers were purchased from Olympus (AT240TS, Oxford Instruments, Abingdon, UK). The probes had nominal spring constants of 2 N/m and driving frequencies of 70 kHz. Images, with a surface from 1 μm^2^, were scanned and the sizes of particles were measured using a free tool in the Asylum Research offline section analysis software.

### 2.11. Supported Lipid Bilayer (SLB) Preparation

To prepare small unilamellar vesicles (SUVs), chloroform solution of POPC (5 mg/mL) and NBD-PE (1% *w*/*v*) were added to a round-bottom flask. The solvent was evaporated under a gentle N_2_ flow to form a uniform thin film on the flask wall. The dried lipid film was emulsified using 10 mM PBS at room temperature and vortexed, to obtain a lipid vesicles dispersion. This latter was extruded through a 100 nm polycarbonate membrane (13 times), followed by another 13 times through a 30 nm membrane (Avanti Polar Lipids). The obtained SUVs were stored under N_2_ and used within 2 weeks, according to the established protocol [25].

### 2.12. Quartz Crystal Microbalance with Dissipation Monitoring (QCM-D)

QCM-D sensor crystals (5 MHz) sputter-coated with 50 nm thin silicon oxide were purchased from QSense, Biolin Scientific, Finland. QCM-D measurements were carried out in flow mode (100 µL/min) by using a Q-Sense instrument (Biolin Scientific, Espoo, Finland). The sensor crystals were cleaned by immersion overnight in a sodium dodecyl sulfate solution (0.4% *w*/*v* in MilliQ water), followed by MilliQ water washing, N_2_ blow drying and UV-ozone treatment for the removal of surface organic contamination (two steps of 15 min each with water rinsing in between and at the end). An additional cleaning was performed by two following steps of rinsing with MilliQ water and PBS in the QCM-D measurement chamber. The fundamental frequency (*n* = 1, 5 MHz) and its odd harmonics (*n* = 3, 5, 7, 9, 11, 13) were monitored as a function of time and the frequency shifts were normalized by dividing for the overtone number. The dissipation changes were also recorded as a function of time.

In the QCM-D experiment, quartz crystal sensor oscillates at the resonance frequency (5 MHz) when an alternating potential is applied. Since the resonance frequency (f) changes upon mass adsorption on the crystal surface, when rigid adlayers are formed, the deposited mass can be calculated from the Sauerbrey’s equation, Equation (7) [61].

(7) $$\Delta m=\;\frac{C}{n}\cdot \Delta f\;$$

where f is the frequency, m the mass adsorbed and n the harmonic number, $C=\;\frac{t_{q}\cdot \rho_{q}}{f_{0}}$, with $t_{q}$ and $\rho_{q}$ being the thickness and the density of quartz, respectively. Such a value corresponds to ~17.7 Hz ng/cm^2^ for a 5 MHz crystal.

### 2.13. Cell Cultures and Treatments

Human Umbilical Vein Endothelial Cells, HUVEC (ATTC, PCS-100-010 code) were cultured in Medium200 supplemented with LSGS, 100 U penicillin/0.1 mg/mL streptomycin. Cells were grown in tissue-culture treated Corning^®^ flasks (Sigma-Aldrich, St. Louis, MO, USA) under a humidified atmosphere of air/CO_2_ (95:5) at 37 °C in an incubator (Heraeus Hera Cell 150C incubator). For HUVEC treatments, the following concentrations of the different samples were used: ANG (2 × 10^−8^ M), CuSO_4_ (1 × 10^−7^ M); ANG + Cu(II) (copper/protein molar ratio = 5); bare AuNP (8.45 × 10^6^ *NP*/mL, corresponding to [AuNP] = 4.6 × 10^−10^ M), AuNP-ANG hybrids in the absence or presence of CuSO_4_ (1 × 10^−7^ M) (Au-wtANG: 2.77 × 10^6^ *NP*/mL corresponding to [AuNP] = 2.1 × 10^−10^ M; Au-rANG: 2.32 ×10^7^ *NP*/mL corresponding to [AuNP] = 3.1 × 10^−10^ M; Au-S28CANG: 1.96×10^6^ *NP*/mL, corresponding to [AuNP] = 2.1 × 10^−10^ M).

### 2.14. Confocal Microscopy Analysis

Confocal microscopy was executed with an Olympus FV1000 confocal laser scanning microscope (LSM), equipped with diode UV (405 nm, 50 mW), multiline Argon (457 nm, 488 nm, 515 nm, total 30 mW), HeNe(G) (543 nm, 1 mW) and HeNe(R) (633 nm, 1 mW) lasers. An oil immersion objective (60xO PLAPO) and spectral filtering systems were used. The detector gain was fixed at a constant value and images were collected, in sequential mode, randomly all through the area of the well. The image analysis was carried out using Huygens Essential software (by Scientific Volume Imaging B.V.; Hilversum, The Netherlands). The statistical analysis was performed with ImageJ software and Student’s *t*-test.

To perform the experiments, 200 µL of HUVEC suspension in Medium200 supplemented with LSGS were plated at a density of 2 × 10^4^ cells per dish in glass bottom dishes of 22 mm in total diameter (12 mm glass-diameter, WillCo-dish^®^, Willco Wells, B.V.; Amsterdam, The Netherland) and cultured at 37 °C in the incubator (Heraeus Hera Cell 150C incubator) for 24 h. Thereafter, cells were treated for 2 h with the different samples at the concentrations specified in Section 2.13. Finally, cells were stained with nuclear dye Hoechst33342 (1 μg/mL), MitoTracker deep red (2 × 10^−7^ M) and the intracellular copper probe CS1 (1 × 10^−6^ M), and fixed with high purity 2% paraformaldehyde in PBS, pH = 7.3.

### 2.15. Cell Viability (MTT) Assay

To perform the cytotoxicity assay, cells were plated in two different 96-well plates, at a density of 7 × 10^3^ cells per well, in complete medium for 24 h. Afterword, cells were treated for 24 h at the concentrations specified in Section 2.13. Cytotoxicity was determined by using the tetrazolium dye 3-(4,5-dimethylthiazol-2-yl)-2,5-diphenyltetrazolium bromide (MTT) [62]. After 2 h of incubation at 37 °C, the enzymatic reduction of MTT to the insoluble purple formazan product was detected by dissolving the crystals with 100 μL of dimethyl sulphoxide and thus measuring the absorbance at 569 nm by Varioscan spectrophotometer. The experiments were performed in triplicate and the results are presented as the means ± SEM.

### 2.16. Scratch Wound Closure Assay

HUVEC cells were seeded and cultured in growth medium until confluence, in 24-well TPP^®^ tissue culture plates coated with 2% *w*/*v* gelatine. The confluent HUVEC monolayers were scratched and wounded using a universal sterile 200-μL pipette tip and then rinsed with complete medium. Plates were marked immediately after the scratch to ensure that scratches were measured at the same location throughout each experiment. Each well was treated with the different samples at the concentrations specified in Section 2.13 at a maximum of incubation time of 24 h. Serial phase contrast images (Leica) of the in vitro wounds were taken immediately after the treatment and then after 5 h and 24 h of incubation. The width of the separation wall was measured by using the MRI Wound Healing Tool on the ImageJ software (version 1.50i, NIH).

### 2.17. Tube Formation Assay

To perform the tube formation assay, Matrigel matrix (Corning, NY, USA) was thawed at 5 °C overnight and spread over each well (50 μL) of a 96-well plate. The plate was then incubated for 30 min at 37 °C to allow the gel to solidify. Thereafter, HUVEC cells (25,000/well) from passage 3 were seeded in 100 μL of Medium 200 supplemented with LSGS at the FBS concentration of 1% (*v*/*v*). After 2 h of incubation at 37 °C with the different samples at the concentrations specified in Section 2.13, the tube structures were observed with a Leika microscope equipped with a digital camera and three bright field images (magnification 4×) were captured for each sample.

## 3. Results

The three proteins, namely wtANG, rANG and the mutant S28CANG were firstly scrutinized in terms of structural conformation and enzymatic activity in order to verify that the introduced the 28 Ser to Cys mutation, in the new protein mutant S28CANG, did not change the secondary structure and the biological function of ANG.

The CD spectra, carried out for the solutions of the three free proteins as well as their complexes with copper ions, showed also for S28CANG the typical CD spectrum of a secondary structure rich in β-strands, with the same distinctive broad negative band centred at around 210 nm of wavelength found both in wtANG and rANG [63,64] (see Supplementary Materials, Figure S1). Additionally for the S28CANG:Cu(II) copper complex, the presence of copper ions, either at equimolar (1:1) or excess molar (1:2) ratio with respect to the protein, did not significantly change the conformation of the mutated protein, similarly to what observed for both wild type and recombinant protein upon the ANG-copper complex formation [65] (Figure S1).

**Figure 1:**
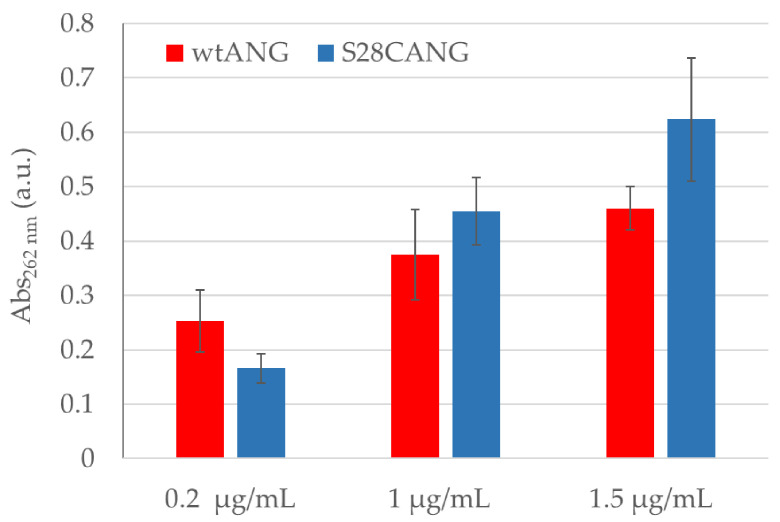
Ribonucleolytic activity of wtANG and S28CANG. Assay was performed at increasing protein concentration (0.2, 0.5, 1, 1.5 μg/mL), in 33 mM MOPS buffer, at 37 °C for 2 h, with the addition of 6 × 10^2^ μg/mL tRNA. Averaged values and standard deviation from three experiments.

The catalytic ability of S28CANG, measured by the RNase assay, increased with the concentration of the protein, with a comparable trend (i.e.; no statistically significantly difference) to that observed for wtANG (Figure 1).

**Figure 2:**
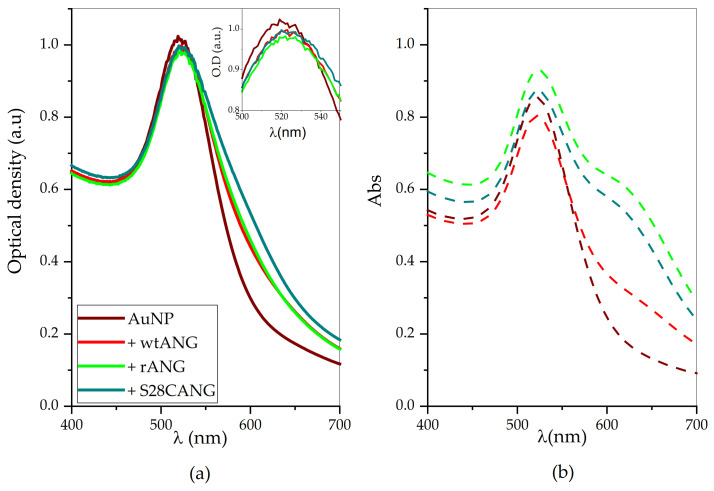
(**a**) UV-visible spectra of (**a**) 8.24 × 10^8^ *NP*/mL AuNPs in 1 mM MOPS (pH = 7.4) before (wine) and after the addition of 5 × 10^−8^ M of ANG (red = wtANG; green = rANG; cyan = S28CANG). In the inset a magnification of the plasmon peak is shown. (**b**) UV-visible spectra of the pellets collected after the washing procedure and re-suspended in MOPS buffer (dashed lines) for bare AuNP and hybrids AuNP-ANG.

Figure 2 shows the optical spectra of the gold nanoparticle dispersion before and after the addition of the three variants of ANG, namely wtANG, rANG and S28CANG. As expected for monodisperse spherical AuNPs with a diameter of about 14 nm, the bare AuNPs show an intense absorption band in the visible region centered at 520 nm [66], and a full width at half maximum (FWHM) value of about 54 nm [67]. For all three cases, the protein addition induces a change of the plasmon peak, with a small increase in the FWHM (2 nm for Au-wtANG; 6 nm for Au-rANG and 8 nm for Au-S28CANG) and red shifts (∆λ ~ 3 nm for both wtANG and rANG; 4 nm for S28CANG), respectively. No significant changes in the maximum of absorbance are instead observed (∆A ~ −0.06 for both wtANG and rANG; ∆A ~ −0.07 for S28CANG) (Table 3).

The UV-vis spectra of the AuNP/ANG hybrids after two rinsing steps (Figure 2b, dashed lines) show that the distinctive plasmon peak shift is maintained (Δλ ~ 2 nm for Au-wtANG and 3 nm for both Au-rANG and Au-S28CANG; ΔFWHM ~ 2 nm, 6 nm, 8 nm for Au-wtANG, Au-rANG and Au-S28CANG, see Table 4) in comparison to the bare AuNPs. However, a shoulder at around 620 nm of wavelength is evident, indicative to the occurrence of nanoparticle aggregation to some extent owing to the washing steps. This effect is especially evident for Au-rANG (in green) and Au-S28CANG (in cyan).

**Figure 3:**
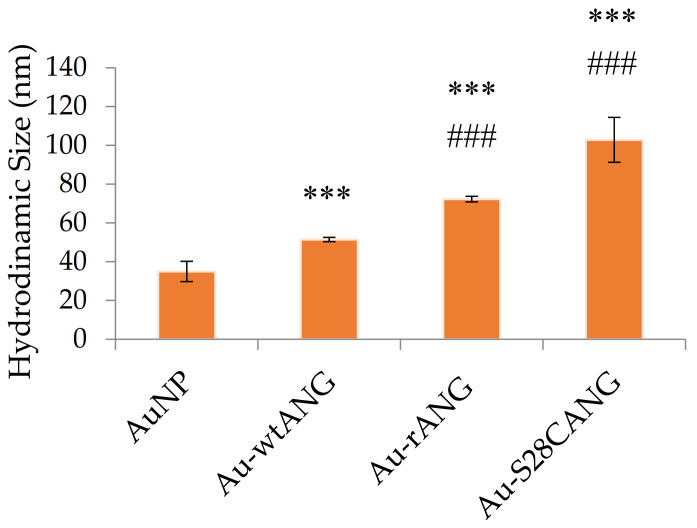
Hydrodynamic size of the bare AuNP (4.3 × 10^8^ *NP*/mL) and the functionalized nanosystems Au-wtANG (2.9 × 10^7^ *NP*/mL), Au-rANG (1.4 × 10^7^ *NP*/mL), Au-S28CANG (7.3 × 10^6^ *NP*/mL), measured by DLS. (***) = *p* < 0.001 vs. AuNP; (###) = *p* < 0.001 vs. Au-wtANG (one-way ANOVA).

DLS measures were carried out on the synthetized hybrid systems to assess the hydrodynamic diameter, which depend on both the single particle and/or clusters of aggregates “core” size and the proteins and solvation shell on the nanoparticles surface. Figure 3 shows, in agreement with the UV-visible spectra, that the measured hydrodynamic size for uncoated AuNPs is 35 ± 5 nm, while the particles size increases up respectively to 51 ± 1 nm for Au-wtANG, to 72 ± 1 for Au-rANG and to 103 ± 12 nm for Au-S28CANG.

**Figure 4:**
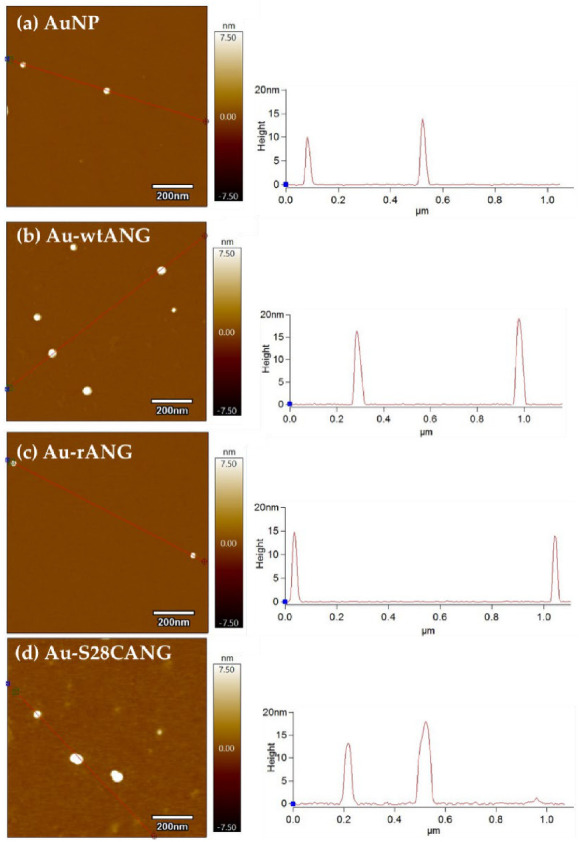
Atomic force microscopy micrographs of (**a**) bare AuNP, (**b**) Au-wtANG, (**c**) Au-rANG and (**d**) Au-S28CANG pellets; scale bar = 200 nm. The graphs on the right of each image show the histograms calculated for Z max.

The bare nanosystems and the protein-functionalized hybrids were characterized by AFM analysis carried out in AC mode in air (Figure 4). The maximum Z-averaged value (nanoparticle height) for the bare gold nanoparticles core is 12 ± 2 nm, according to the UV-vis spectra discussed above. Whereas, for the washed pellets of Au-wtANG, Au-rANG and Au-S28CANG, the measured values were respectively of 17 ± 2 nm, 14 ± 1 nm and 18 ± 2 nm.

QCM-D experiments were performed to investigate the influence of the protein shell surrounding the functionalized nanosystems, in the interaction with model cell membranes made of supported lipid bilayers, in comparison with the bare AuNPs. Besides the deposited mass, QCM-D allows for monitoring a second parameter related to the energy loss, the dissipation (D), turning off the driving potential and monitoring the decay of the crystal oscillations. Such a parameter gives meaningful information about the viscoelastic properties of the film adsorbed, which are useful to characterize the interaction of SLBs with different molecules and nanosystems [68].

**Figure 5:**
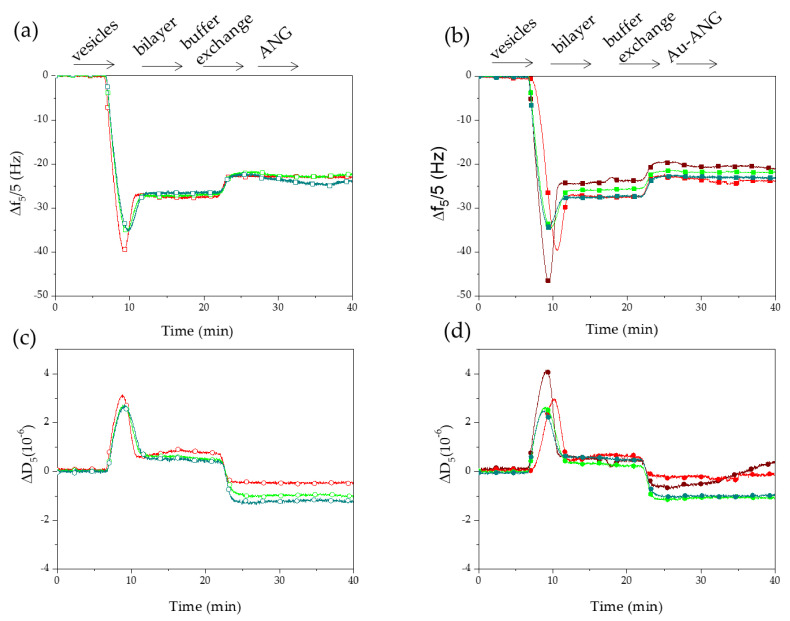
QCM-D curves of frequency (**a**,**b**) and dissipation (**c**,**d**) shifts corresponding to the fifth overtones (*n* = 5) for POPC SUVs adsorption followed by the addition of 5 × 10^−8^ M free proteins (open symbols; wtANG = red; rANG = green; S28CANG = dark cyan) or AuNP-based systems (solid symbols; 2.3 × 10^7^ *NP*/mL citrate-capped bare nanoparticles = wine; 2.5 × 10^6^ *NP*/mL Au-wtANG = red; 1.6 × 10^6^ *NP*/mL Au-rANG = green; 1.6 × 10^6^ *NP*/mL Au-S28CANG = dark cyan. Experiments were performed in 1 mM MOPS buffer (pH = 7.4).

Figure 5 shows the QCM-D curves of Δf and ΔD after the adsorption of zwitterionic SUVs made of POPC-NBD, followed by the addition of 2.3 × 10^7^ *NP*/mL (3 × 10^−9^ M) bare AuNP, 2.5 × 10^6^ *NP*/mL (9 × 10^−10^ M) Au-wtANG, 1.6 × 10^6^ *NP*/mL (9 × 10^−10^ M) Au-rANG, 1.6 × 10^6^ *NP*/mL (9 × 10^−10^ M) Au-S28CANG, and the proteins at the concentration of 5 × 10^−8^ M wtANG and S28CANG, and 2 × 10^−8^ M rANG, in 1 mM MOPS buffer solution.

The first part of the experiment displays the typical two-phase process expected after the addition of POPC SUVs on the SiO_2_-coated crystals. The first phase reflects the adsorption of the vesicles on the silica surface until the minimum in frequency shift (increase in coupled mass) and the maximum in dissipation shift (viscoelastic properties of the vesicles) are reached. This point corresponds to a critical coverage of the intact vesicles on the SiO_2_ surface. The second phase reflects the vesicles disruption followed by the formation of a regular SLB. Typical values of Δf ~ −26 Hz and ΔD < 0.5 × 10^−6^ indicate the formation of a homogeneous and stable lipid bilayer, in agreement with literature data [69].

The next step of solvent exchange, from PBS to MOPS buffer, exhibits shifts in both the curves of frequency and dissipation, due to density and viscosity changes of the liquid layer at the interface with the sensor. Upon the addition of free ANG proteins (Figure 5a–c), no significant perturbation of the membrane was detected (i.e.; no significant shifts in frequency and/or dissipation) except in the case of the mutant S28CANG, which induces a slight shift of the frequency of about −2.02 Hz. Another significant outcome of the QCM-D experiments was that by adding the bare citrate-capped AuNPs to the pre-formed SLB a significant perturbation of the membrane was induced, as displayed by the shift $\Delta D\sim 1\times 10^{-6}$ in the dissipation curve. On the other hand, this effect was not evident for the addition of the three AuNP-ANG hybrids. Such a finding further confirms the full coverage of the gold nanoparticles in the case of the three studied proteins.

The response of endothelial cells to the hybrids as well as to the bare AuNPs and to the free proteins, used as positive controls, was investigated in terms of cytotoxicity through MTT assay, cell migration capability and angiogenic activity through wound scratch and tube formation assays, respectively, as well as cellular internalization and interaction with intracellular organelles by mean of confocal microscopy analysis.

**Figure 6:**
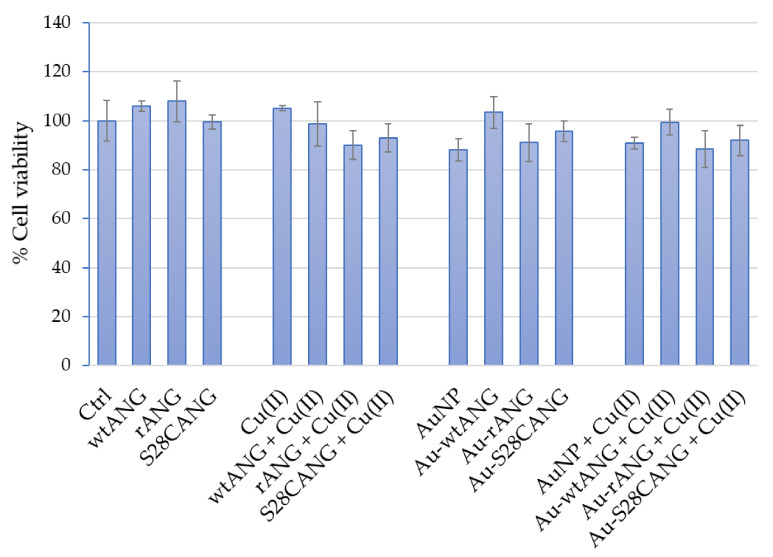
Cell viability assay (MTT) on HUVECs untreated (negative control) or treated for 24 h with: 20 nM free ANG proteins; 100 nM CuSO_4_; ANG—copper complexes ([Cu(II)]/[ANG] = 5); bare AuNP and AuNP-ANG hybrids, in the absence or presence of 100 nM CuSO_4_. The following nanoparticle concentrations were used: 0.5 nM AuNP (8.45 × 10^6^ *NP*/mL); 0.2 nM Au-wtANG (2.77 × 10^6^ *NP*/mL); 0.3 nM Au-rANG (2.32 × 10^7^ *NP*/mL); 0.2 nM Au-S28CANG (1.96 × 10^6^ *NP*/mL). The bars represent means ± S.D. of three independent experiments performed in triplicate (S.D. = standard deviation).

MTT assay (Figure 6) was carried out on HUVEC cells treated for 24 h with the different samples at the concentrations specified in Section 2.12. The cell incubation with free proteins, namely wtANG, rANG, S28CANG did not induce any significant change on cell viability with respect to the untreated cells; similar results were found for cells treated with the proteins in the presence of copper, even if a small reduction of cell viability (<10 ± 6%), is observed with respect to the proteins alone. As to nanoparticle-treated cells, the incubation with bare AuNP, both in the absence and with Cu(II), led to a small (<10 ± 4%), but no statistically significant reduction of the cell viability in comparison with control cells. As to the hybrids alone and in medium supplemented with copper ions, the obtained results demonstrated their high biocompatibility not showing any significant decrease of cell viability in comparison to untreated control cells. Dose-response experiments of viability confirmed the same trend (see Figure S2 in Supplementary Information).

Wound closure and tubulogenesis assays were carried out to study the effects respectively on the wound repair and the sprouting capability of the synthetized systems, both in the absence or presence of copper ions.

**Figure 7:**
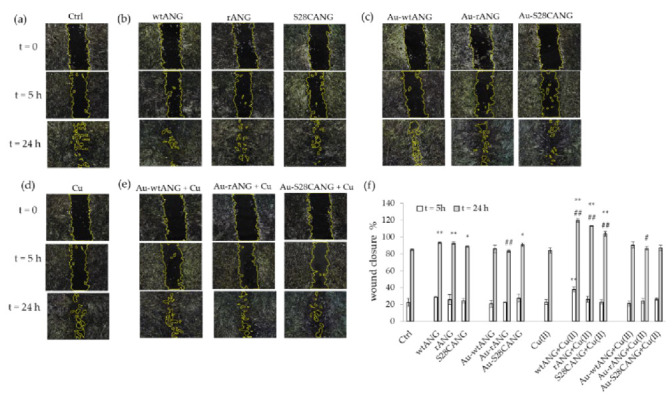
Representative micrographs of HUVECs, untreated (**a**): negative control) or treated with the different samples at time 0, 5 h and 24 h after scratch: (**b**) 20 nM free ANG proteins; (**c**) AuNP-ANG hybrids in the absence of copper; (**d**) 100 nM CuSO_4_; (**e**) AuNP-ANG hybrids in the presence of 100 nM CuSO_4_. The following nanoparticle concentrations were used: 0.2 nM Au-wtANG (2.77 × 10^6^ *NP*/mL); 0.3 nM Au-rANG (2.32 × 10^7^ *NP*/mL); 0.2 nM Au-S28CANG (1.96 × 10^6^ *NP*/mL). In (**f**): quantitative analysis of migration assay (wound edge advancement in percent vs. time). Values (means ± SEM) are from three independent experiments. Statistical analysis was performed by pairwise Student’s *t*-test. (*) *p* < 0.05, (**) *p* < 0.01 vs. CTRL; (#) *p* < 0.05, (##) *p* < 0.01 vs. the corresponding free ANG protein. Results are expressed as percentage of wound closure with respect to time 0.

**Figure 8:**
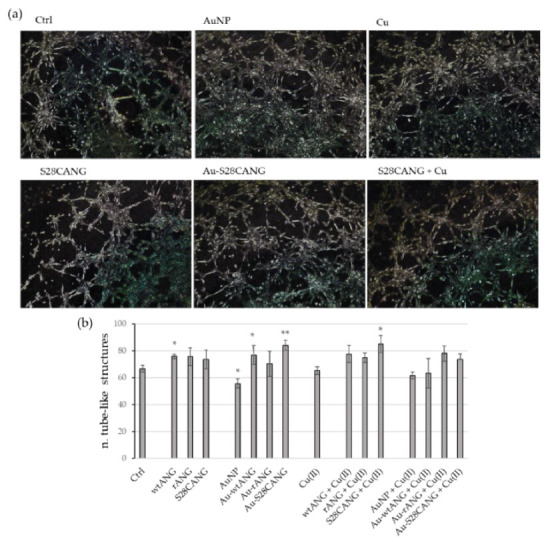
Tube formation assay. (**a**) Representative bright-field optical images of HUVEC cells cultured on Matrigel matrix, untreated and treated for 5 h in M200 supplemented with 1% *v/v* FBS with: 20 nM free ANG proteins; 100 nM CuSO_4_; ANG—copper complexes ([Cu(II)]/[ANG] = 5); bare AuNP and AuNP-ANG hybrids, either in the absence or presence of 100 nM CuSO_4_. The following nanoparticle concentrations were used: 0.5 nM AuNP (8.45 × 10^6^ *NP*/mL); 0.2 nM Au-wtANG (2.77 × 10^6^ *NP*/mL); 0.3 nM Au-rANG (2.32 × 10^7^ *NP*/mL); 0.2 nM Au-S28CANG (1.96 × 10^6^ *NP*/mL). (**b**) The number of closed networks of vessel-like tubes was counted from three experiments. Data are mean ± S.D. (*) *p* < 0.05, (**) *p* < 0.01 vs. CTRL (Student’s *t*-test).

The quantitative analysis of the wound scratch test (Figure 7), showed the treatments with the three ANG + Cu(II) samples as the most effective to enhance the cellular migration. On the other hand, as displayed in Figure 8, the Matrigel test pointed to the highest response in terms of tube formation for the cells treated with Au-S28CANG and S28CANG + Cu(II).

**Figure 9:**
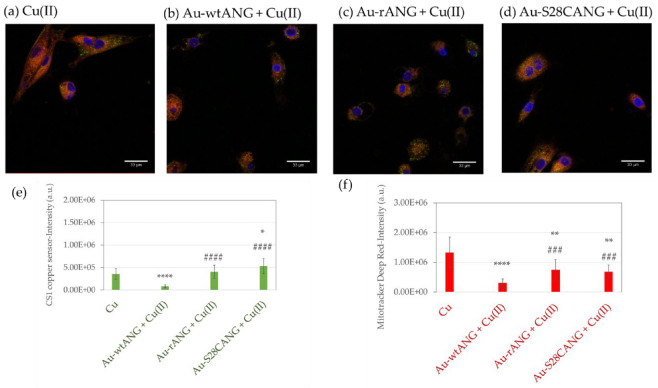
LSM micrographs of HUVECs (in blue, nuclear staining, λex/em = 405/425–450 nm; in green, CS1-copper probe, λex/em = 543/550–600 nm, in red mitochondrial staining, MitoTracker d.r λex/em = 633/650–655 nm) after 2 h of treatment with: (**a**) CuSO_4_ (1 × 10^−7^ M); (**b**–**d**) AuNP-ANG hybrids in the presence of CuSO_4_ (1 × 10^−7^ M) (Au-wtANG: 2.77 × 10^6^ *NP*/mL corresponding to [AuNP] = 2.1 × 10^−10^ M; Au-rANG: 2.32 × 10^7^ *NP*/mL corresponding to [AuNP] = 3.1 × 10^−10^ M; Au-S28CANG: 1.96 × 10^6^ *NP*/mL, corresponding to [AuNP] = 2.1 × 10^−10^ M). Scale bar 10 μm. Intensity value of fluorescence corresponding to the (**e**) CS1 copper probe and (**f**) Mitotracker deep red staining. Bars represent means ± SEM of at least 3 experiments; (*) = *p* < 0.05, (**) = *p* < 0.01, (****) = *p* < 0.0001 vs. Cu; (###) = *p* < 0.01, (####) = *p* < 0.0001 vs. Au-wtANG (Student’s *t*-test).

Noteworthy, the cellular treatment with the Au-S28CANG resulted also the most effective to trigger the intracellular copper influx (Figure 9). In particular, LSM intracellular imaging was carried out to evaluate: (i) the cellular uptake of AuNPs functionalized with the three studied proteins; (ii) to image the perturbation on the copper trafficking and mitochondria. All the samples treated with the protein-functionalized gold nanoparticles showed small aggregates of nanoparticles inside the cells, visible as dark dots, thus proving the effective entrance of the nanosystems within the endothelial cells (see Figure S3 in Supplementary Information). Larger aggregates were found for the hybrids Au-wtANG and Au-S28CANG, mostly confined at the cellular membrane as expected because of the ability of the physiological ANG to bind to the cytoskeletal actin.

As to the intracellular Cu(I) staining by the CS1 probe, the quantitative analysis (Figure 9e) shows that, with reference to the positive control of copper-treated cells (Figure 9a), a significant reduction of copper was detected for the HUVECs, after the treatment with Au-wtANG + Cu(II) (Figure 9b), no significant differences were displayed by cell treatment with Au-rANG + Cu(II) (Figure 9c) and a small, but statistically significant increase of intracellular copper for the samples incubated with Au-S28CANG + Cu(II) (Figure 9d) was found. As to the interaction with mitochondria, in general all the treatments with the functionalized nanosystems affected the mitochondrial activity leading to their disfunctions as evidenced by the reduction of the mitochondrial staining in comparison with the control (Figure 9f).

## 4. Discussion

The angiogenin protein, a 14.2-kDa plasma protein, was anchored to 14 nm-diameter gold nanoparticles, in order to create hybrid nanoplatforms, combining the antioxidant and anti-angiogenic action of the colloidal gold to the biological activity of the protein, for advanced wound care applications.

The plasmonic properties of gold nanoparticles allowed to discriminate among the different binding modes of the three ANG proteins used at interface with the metal nanoparticle, through a mechanism of prevalent chemical adsorption (for S28CANG) or physical adsorption (wtANG and rANG), respectively. The surface plasmon excitation process, indeed, is influenced both by chemical and physical effects induced by the surrounding media.

In particular, the chemical interface damping (CID) effects on plasmonic changes involves chemisorption and physisorption of molecules on the particle surface [70]. The adsorption of molecules, providing new relaxation pathways to the free electrons on the metal nanoparticle, leads to a widen and red-shifted plasmon peak, due to the coupling of the metal free conduction electrons with the unoccupied molecular orbitals of the absorbed molecules on the particle surface [70]. The dielectric properties of the environment or the ligand layer upon the nanoparticle surface also modifies the plasmon peak in its absorbance maximum, width and intensity. In particular, surrounding medium with a high refractive index as well as nanoparticle coating layer with high polarizability (e.g.; protein [71]) cause a gain of the SPR intensity, a red-shift and an increase of the full width at half maximum (FWHM) value of the plasmon peak [72].

A new variant of ANG was inspected, i.e.; S28CANG, with a cysteine instead of the serine at the residue 28, for the high affinity binding to the metal surface, through Au-S bonds.

Gold, indeed, being a soft acid, binds to soft bases like thiols, to form stable Au-S bonds (40–50 kcal/mol) that are able to replace the citrate shell on the nanoparticle surface due to the strong binding affinity of the thiol groups to the metal. Therefore, in the case of the mutant protein S28CANG, a predominant chemisorption mechanism is expected at the interface with AuNPs, while a pure physisorption process would occur for both wtANG and rANG, respectively.

Since copper is another crucial angiogenic factor, the interaction with copper ions of the new mutant S28CANG was scrutinized in the comparison with wtANG and rANG.

Among the cytokines, ANG binds to 2.4 mol of copper ions per mol of protein at physiological pH [73]. Such an interaction involves, preferentially, the receptor binding site of the protein and affects the biological function of the protein [63]. Previous works reported that the complex formation between divalent copper and ANG decreases the nuclear translocation of the protein and its ribonucleolytic activity [74]. Moreover, in the presence of copper ions, the interaction between ANG and endothelial cells increases [73]. As stated above, rANG, expressed into bacterial vectors and typically used for research works, contains an extra methionine residue at the N-terminal domain. On the other hand, wtANG, which is physiologically presents in human plasma, shows the glutamine, as first residue, which spontaneously cyclized to a pyroglutamate ring. This structural variation, between recombinant and wild-type, affects the copper complex formation and the metal coordination environment. Thus, rANG mainly binds to Cu(II) through the free terminal amino group, under physiological conditions. Instead, in the wtANG, being the amino terminal group locked, the His114 residue, which is one of the three essential amino acids responsible for the RNase activity of the protein, is primarily involved in the Cu(II) complex formation, under physiological conditions [65]. Such a relevant difference between rANG and wtANG in the copper binding would influence the biological properties of the proteins in the presence of copper ions.

The RNAse activity of the new mutant (Figure 1) was confirmed comparable with that of the wtANG, as well as the secondary structure, as demonstrated by CD analyses (Figure S1).

The successful ANG proteins immobilization at the surface of AuNP was detected by the plasmonic changes (Figure 2), by the red-shift in the wavelength of maximum absorption (λ_max_) as well as a broadening in the FWHM of the plasmon peak for the protein-added nanoparticles in comparison to bare AuNPs.

The peak broadening is related to an increase of the polydispersity of the colloidal suspension, due to the presence of several size populations of nanoparticles associated with aggregation effects [75,76] and/or the effective size increase upon the molecule adsorption at the surface of the nanoparticle [77].

As to the aggregation, this effect cannot be ruled out because, upon the interaction between the positively charged groups (lysine, arginine, histidine as well as the free N-terminus group for rANG) of the protein at the physiological pH and the citrate-capped negatively charged AuNPs, the electrostatic stabilization mechanism of the colloidal dispersion is expected to be partially or even totally nullified, with consequent polydispersity of the suspension [78]. On the other hand, the steric stabilization effect is expected to reduce the polydispersity contribution due to aggregation and, at the same time, to rise that due to the actual increase of nanoparticle diameter (i.e.; metal core + protein shell). The nanoparticle size increase calculated from the optical spectra (Table 3 and Table 4) confirmed the successful immobilization of all three proteins onto the AuNPs.

As to the adsorption of molecules on the nanoparticle surface, as expected, it induces variations on the local dielectric environment, manifested as changes into λ_max_ and the peak absorbance (A_max_). To this respect, one can use theoretical calculations to calculate the nanoparticle coverage by taking into account how the local refractive index environment of the particle affects the plasmonic peak [79]. In particular, by assuming the peptide-coated nanoparticles as core-shell spheres with a metallic core of d_opt_ diameter, corresponding to the uncoated nanoparticles, and a homogeneous spherical proteinaceous shell, the fraction of protein over the total particle, g, is related to the changes of $\lambda_{max}$, as given by the following Equation (8):

(8) $$g=\left(1+\alpha_{s}\right){\left(\frac{\lambda_{p}^{2}\left(\varepsilon_{s}-\varepsilon_{m}\right)}{\Delta \lambda \cdot \lambda_{max,0}}+2\alpha_{s}\right)}^{-1}$$

where $\lambda_{p}$ is the free electron oscillation wavelength (which is 131 nm for gold [80]), ε is a dielectric constant or relative permittivity (equal to the squared refractive index), the ‘s’ and ‘m’ subscripts refer to the shell and surrounding medium, respectively; $\lambda_{max,0}$ is the wavelength of maximum absorption for uncoated colloid and $\alpha_{s}=\frac{\left(\varepsilon_{s}-\varepsilon_{m}\right)}{\left(\varepsilon_{s}+2\varepsilon_{m}\right)}\;$is the polarizability of a sphere of shell dielectric constant $\varepsilon_{s}$ in a medium of dielectric constant $\varepsilon_{m}$. By considering the refractive index values (n) at 550 nm respectively of 1.335 for water [81] and 1.38 for a pure protein [82], a protein fraction shell of g=0.74 for Au-wtANG and Au-rANG and g=0.97 for Au-S28CAng, could be calculated.

From the g value and according to the following equation Equation (9), which refers to the shell thickness (s):

(9) $$s=\frac{d}{2}\left[\frac{1}{{\left(1-g\right)}^{1/3}}-1\right]$$

and using the Feijter’s formula in the equation below Equation (9):

(10) $$\Gamma =s\frac{n_{s}-n_{m}}{dn/dc}$$

where, Γ is the coverage and dn/dc is the refractive index increment (typically 0.19 mL⋅g^−1^ for a protein [83]), the calculated mass of protein absorbed on the synthetized hybrid Au-protein per unit area is about 80 ng/cm^2^ for Au-wtANG and Au-rANG, and of 320 ng/cm^2^ for Au-S28CANG, respectively. Thus, given the molecular weight of ANG (MW = 14,200 g/mol) and the diameter *d* = 14 nm for each spherical AuNP, the corresponding Γ in terms of molecular coverage is calculated as follows: 20.9 molecules of wtANG (or rANG) and 83 molecules of S28CANG per each NP. Since the average angiogenin molecular dimensions are (70 × 62 × 32) Å^3^ [84] the ideal monolayer coverage of the protein in the two end-on and side-on limit configurations is about 31 and 14 molecules/nanoparticle, respectively. Accordingly, at nanoparticle surface it can be figured out a bilayer coverage of wtANG, rANG and a multilayer of S28CANG, most likely in the end-on configuration.

As to the pellets recovered after the washing steps, according to the Equations (8)–(10), for Au-wtANG pellet 2 (∆λ ~ 2 nm), the theoretical protein fraction shell is g = 0.5, corresponding to a coating thickness of s = 1.54 nm and a mass of protein absorbed of Γ = 36 ng/cm^2^. On the other hand, for both Au-rANG and Au-S28CANG pellets 2 (∆λ ~ 3 nm), the calculated values are: g = 0.74, s = 3.36 nm and Γ = 79 ng/cm^2^. Therefore, the washing steps cause a loss of unbound and/or weakly bound proteins respectively of about 55% for Au-wtANG and 75% for Au-S28CAng. As to Au-rANG, apparently no significant loss of protein is detected since the shift in the wavelength at the maximum absorbance is similar to that before the nanoparticle rinsing (∆λ ~ 3 nm). These findings suggest that different adsorption modes are expected for the cysteine-mutated protein with respect to the wild type and to the recombinant angiogenin.

As to the Au-S28CAng, indeed, the substitution of the serine with the cysteine residue, leads to a prevalent chemisorption process, involving the formation of strong thiol-gold bonds, which further trigger “ordered” domains of protein around the nanoparticle [85]. On the contrary, both wtANG and rANG interact with the AuNP surface by a physisorption process, leading to a “disordered” molecular layer at the nanoparticles interface. However, in the case of rANG, the presence of an extra positively charged group at the N-terminal methionine residue can drive a weaker immobilisation of the protein molecules at the interface with the gold surfaces in comparison with wtANG. Hence, after the rinsing, more homogeneous ”protein patches” at the AuNP surface are expected for Au-wtANG than Au-rANG. It is postulated that a ‘disordered’ protein coating of gold nanoparticles can contribute to the optical spectra in a similar way than a thick shell; accordingly [79], the use of Equations (4)–(6) for Au-rANG may result in an overestimation of the actual protein coverage [86].

The same trend of optical size change was observed in the hydrodynamic size, determined by DLS (Figure 3), with a measured increase of the hydrodynamic diameter for the AuNP-ANG hybrids with respect to the bare AuNP. In particular, DLS also pointed out to the parallel occurrence of both nanoparticle aggregation and nanoparticle size increase (due to the effectiveness of protein surface immobilization at the nanoparticle surface). In particular, evidence on the presence of big cluster of nanogold aggregate together with the AuNP-protein hybrids was found for the Au-rANG and Au-S28CANG samples, in agreement with UV-visible spectra (Figure 2), which showed for these systems a shoulder at around 620 nm in the plasmon peak, especially evident after the washing procedure (Figure 2b).

Accordingly, AFM analyses (Figure 4) showed an increase in the particle size for the AuNP-ANG hybrids with respect to bare AuNPs, thus confirming the effective surface decoration of the gold surface by the three proteins, with different arrangement modes. The shrinking of the size measured by AFM compared to the size measured/calculated in liquid, i.e.; respectively by DLS/UV visible spectroscopy, is explained in terms of a ‘collapse’ of the protein layer physisorbed and/or chemisorbed (as in the case of S28CANG) onto the nanoparticle surface owing to the dewetting during the AFM samples drying.

The effective immobilization of the proteins on the AuNP to obtain Au-ANG hybrids was also proved by QCM-D experiments (Figure 5). SLBs were used in these experiments as artificial model of cell membrane, to study their interaction with the free proteins (Figure 5a,c) as well as with the bare (AuNP) or the protein-functionalized (AuNP-ANG) gold nanoparticles (Figure 5b,d). The addition of the free proteins to the pre-formed SLB did not significantly change the QCM-D curves of frequency and dissipation, except than for the mutant S28CANG, which induced a frequency shift of about −2.02 Hz. In the assumption of a rigid adlayer, such a finding points to an adsorbed mass of about 40 ng/cm^2^. This effect can be due to the fact the cysteine is more hydrophobic than serine, thus S28CANG has a higher affinity to bind the phospholipid bilayer than wtANG and rANG. As to the bare AuNP, the interaction with SLB produced no significant changes in the frequency but a large shift of dissipation ($\Delta D\sim 1\cdot 10^{-6}$), which pointed to significant modifications of the viscoelastic properties of the model membrane systems [87]. Interestingly, in the case of AuNP-ANG samples, no significant shifts were detected in all three cases, thus confirming that the cysteine residues in S28CANG were effectively involved in the interaction with the metal surface of the nanoparticle, hence resulting buried at the outermost surface of the hybrid nanosystem.

The hybrid AuNP-ANG assemblies were used as a multifaceted nanoplatform to modulate the balance between the naturally secreted pro- and anti-angiogenic factors by combining the angiogenic properties of angiogenin protein and copper ions, to the anti-angiogenic properties and antioxidant activity of gold nanoparticles, for potential wound healing application. The nanoplatform, other than combining the above-mentioned intrinsic proprieties of each component, offers the advantage of an improved selective delivery of the protein as well as enhanced therapeutic outcomes by increasing the membrane permeability of ANG, limiting its endosomal escape and the enzymatic degradation, thereby leading to a prolonged half-life of the protein [88].

To test the cell viability as well as the angiogenic response in terms of cell migration and tubulogenesis activity in vitro cellular experiments on endothelial cells were carried out.

As to cytotoxicity, the new mutant S28CANG was found to not affect significantly the HUVECs viability, and the same was confirmed for wtANG and rANG [27,74] at the used experimental conditions (Figure 6 and Figure S2). As to the cellular treatments in copper-supplemented medium, no differences were found with respect to the untreated cells even if a small but no-significant reduction of cell viability was observed for rANG and S28CANG, thus evidencing a weak effect of the protein-copper complex formation in the interaction with the cells. These results are in agreement with literature data which prove that the IC_50_ for Cu(II) in HUVECs is 500 mM [89,90]. As to the nanosystems, it is known that the effect of NPs on the cell viability is closely associated with the NPs composition (e.g.; Au, Pt, Ag), coating and size. In agreements with literature data which demonstrate that citrate capped AuNPs with a size between 10 and 50 nm do not affect the viability of HUVECs in a concentration range 0.1–100 nM [91,92], our results did not evidence cytotoxic effect at the tested conditions. Similarly, the hybrids both in the absence and in the presence of copper did not show any cytotoxicity evidencing the good potentialities of our protein-conjugated NPs as cell specific, angiogenic nanomedicine tools.

Crucial to the healing process is the movement in the wound area of several cell types including white blood cells, dermal fibroblasts and in particular endothelial cells which during the initial stage of angiogenesis were stimulated to elongate and migrate in order to from new lumen-bearing tubes to conduct the blood flow [93]. It is also known that ANG, through the amino acid sequence encompassing the residues from 60 to 68, that are also part of the cell-surface receptor binding site, makes complex with the endothelial cell surface α-actin, thus promoting endothelial cell migration and invasion into the perivascular tissue, which is a crucial phase of the vessel growth [94].

Our results confirmed the prompting of ANG protein action both in cell migration and tubulogenesis. In particular, the wound healing assay demonstrated a comparable response for the cells treated with the hybrid with the new mutant Au-S28CANG with respect to those treated with the free ANG proteins, even if the most effective treatment was that with ANG + Cu(II) (Figure 7). In the case of the Matrigel assay, we found a ameliorative effect with respect to the negative control upon the cell treatment with Au-S28CANG (Figure 8). These findings can be related to the different functionalization approach used for the mutant, namely the chemisorption, that triggered by the Au-S bond formation leads to a more ordered and stable shell of ANG on the nanoparticles surface which provide a more specific and effective interaction with the cells [95]. Differently the physisorption process of wtANG and rANG on gold surface seems to reduce the biological role of ANG. This could be due to the random orientation of the proteins at the gold nanoparticle surface and to the electrostatic attractions, which allow they adsorption on the AuNP surface and that could make the biological response difficult to control. Moreover, because proteins are non-covalently conjugated to nanoparticles their interaction is not stable and the protein can be easily replaced by other molecules in the biological medium [40,96]. As to the interaction with copper ions, accordingly to previous works which reported that copper controls and modulates the angiogenic response and the biological function of ANG [97] decreasing both the nuclear translocation of the protein and its ribonucleolytic activity [74], our results show that this metal affects the biological response of the proteins alone, most likely due to small conformational changes induced during the complex formation, as evidenced by the CD spectra (Figure S1), that further change its biological behavior leading to an increase of their cell migration capabilities.

The LSM analyses demonstrated the effective entrance of the nanosystems into the cells and their interaction with mitochondria (Figure 9). Our results, indeed, revealed a significant reduction of the intensity of the mitochondrial staining after the treatment with all the hybrids nanosystems compared to the positive control (Figure 9f), in agreement with literature data which demonstrated that AuNP by interacting with mitochondria can cause their dysfunction with loss of membrane potential [98,99]. Interestingly, the cell treatment with Au-S28CANG + Cu(II) significantly increased the intracellular copper in HUVECs with respect to the treatment with Au-rANG + Cu(II) (comparable levels of monovalent copper detected than the positive control of cells treated with CuSO_4_) and Au-wtANG + Cu(II) (lower amount of intracellular copper detected with respect to the positive control). These findings pointed to the promising potentiality of the Au-S28CANG as nanoplatform for drug delivery, evidenced by the enhanced ability of the S28CANG adsorbed on the gold surface to strongly bind copper ions, thus allowing for trafficking the metal inside the endothelial cells.

## 5. Conclusions

In this work, hybrid nanosystems were fabricated to specifically modulate the angiogenic process with potential application in wound care. In particular, gold nanoparticles were used as an anti-angiogenic component with ROS quencher (hence anti-inflammatory) capability of a hybrid nanocomposite with the angiogenin protein (pro-angiogenic), in order to modulate the angiogenic processes involved in the wound healing.

A newly expressed mutant (S28CANG) was specifically designed to introduce a thiol-free cysteine residue on the protein, to compare the prevalent chemisorption effect at the surface of the gold nanoparticle to the pure physisorption expected for both the wild type (wtANG) and the recombinant (rANG) forms. A “disordered” shell of protein molecules was figured out around the nanoparticle surface in the Au-wtANG and Au-rANG hybrid nanoplatforms, while an “ordered” multi-layered arrangement of molecules around the AuNP core was identified for the chemisorbed systems, due to formation of the strong thiol-gold bonds.

The interaction of the protein-decorated gold nanoparticles at the bio-interface with an artificial model of cell membranes (supported lipid bilayer) and with human umbilical vein endothelial cells was scrutinized. The QCM-D studies on the interaction between the protein-decorated gold nanoparticles and the SLBs confirmed the effective coating of the nanoparticles by the protein molecules, as well as the capability of the new mutant S28CANG to reproduce the interaction of the physiological wtANG, once immobilized onto the gold nanoparticle surface. Cellular experiments evidenced the high biocompatibility of the hybrids and confirmed the key role of copper as co-player in influencing the biological activity of ANG. These results are very promising to develop a novel multifunctional platform for wound care treatment and tissue regeneration applications.

## Figures and Tables

**Table 1:** Specifications for the PCR steps.

Round	N. of Cycles	Temperature (°C)	Time (s)
1	1	95	120
2	18	95	20
		60	20
		68	480
3	1	68	350

**Table 2:** Calculated concentrations (in mol/L and *NP*/mL) of AuNPs and number and number of ANG molecules per *NP* used for the functionalization protocol.

Sample	[AuNP] mol/L	AuNP/mL	[ANG] mol/L	ANG Molecules/*NP*
Au-wtANG	1.0 × 10^−8^	8.24 × 10^8^	5.0 × 10^−8^	3.7 × 10^4^
Au-rANG	1.0 × 10^−8^	8.24 × 10^8^	2.0 × 10^−8^	1.5 × 10
Au-S28CANG	1.0 × 10^−8^	8.24 × 10^8^	5.0 × 10^−8^	3.7 × 10^4^

**Table 3:** UV-visible quantitative analysis of AuNP-ANG samples before washing. Experimental values of: shifts of the maximum of absorption (∆A_max_), shifts of the wavelength at the absorbance maximum (λ_max_) and full width at half maximum (FWHM). Calculated values of optical diameters.

Sample	ΔA	Δλ (nm)	FWHM (nm)	d_opt_
AuNP	-	-	54	14
Au-wtANG	−0.06	3	56	22
Au-rANG	−0.06	3	60	22
Au-S28CANG	−0.07	4	62	25

**Table 4:** UV-visible quantitative analysis of washed AuNP-ANG samples. Experimental values of maximum of absorption (A_max_), wavelength shift at the absorbance maximum (Δλ) and full width at half maximum (FWHM). Calculated values of extinction coefficient (ε), optical diameter (*d*) and nanoparticle concentration for the pellets re-suspended in 1 mM MOPS.

Pellet Sample	A_max_	Δλ (nm)	FWHM (nm)	ε (cm^−1^M^−1^)	d_opt_	[nM]	[*NP*/mL]
AuNP	0.867	-	54	1.3·10^7^	14	60.9	4.3·10^8^
Au-wtANG	0.803	2	55	7.8·10^8^	19	10.3	2.9 ·10^7^
Au- rANG	0.930	3	60	1.3·10^9^	22	7.4	1.4·10^7^
Au-S28CANG	0.872	3	62	1.3·10^9^	22	6.9	7.3 ·10^6^

## Data Availability

Not Applicable.

## Supplementary Materials

The following are available online at https://www.mdpi.com/2079-4991/11/1/201/s1, Figure S1: Far-UV CD spectra of ANG; Figure S2: Cell viability assay on HUVECs treated for 24 h with ANG samples; Figure S3: Optical bright field micrographs of HUVECs treated for 24 h with the hybrids Au-ANG.
