# Supplementary material for: Gold Nanoparticles Functionalized with Angiogenin for Wound Care Application
Source: Nanomaterials (Basel). 2021 Jan 14;11(1):201. doi: 10.3390/nano11010201 (PMC7830515; doi:10.3390/nano11010201)
Supplement: Supplementary file 1 [file nanomaterials-11-00201-s001.pdf]

nanomaterials

MDPI

Supplementary Information

Gold Nanoparticles Functionalized with Angiogenin for Wound Care Application

Lorena Maria Cucci [1], Giuseppe Trapani [2], Örjan Hansson [3], Diego La Mendola [4,*] and Cristina Satriano [1,*]

[1] Laboratory of Hybrid NanoBioInterfaces (NHBIL), Department of Chemical Sciences, University of Catania, 95125 Catania, Italy; lorena.cucci@unict.it
[2] Scuola Superiore di Catania, University of Catania, 95123 Catania, Italy; giuseppe.trapani@studium.unict.it
[3] Department of Chemistry and Molecular Biology, University of Gothenburg, SE-40530 Göteborg, Sweden; orjan.hansson@chem.gu.se
[4] Department of Pharmacy, University of Pisa, 56126 Pisa, Italy
* Correspondence: lamendola@farm.unipi.it (D.L.M.); cristina.satriano@unict.it (C.S.); Tel. +39-050-2219533 (D.L.M.); +39-095-7385136 (C.S.)

Materials and Methods

Circular Dichroism (CD)

CD spectra were recorded on a Jasco model 810 spectropolarimeter, in the 195-270 nm wavelength region, at RT and under a constant flow of nitrogen. Spectra were obtained at the scan rate of 50 nm/min and a resolution of 1 nm using quartz cuvettes with 0.1 cm optical path length as an average of 10 scans. The recorded spectra of the free proteins (wtANG, rANG and S28CANG) at the concentration of 2 × 10^{-6} M in 1 mM MOPS, before and after the addition of Cu(II) at the ANG:Cu(II) molar ratio of 1:1 and 1:2 are showed in Figure S1.

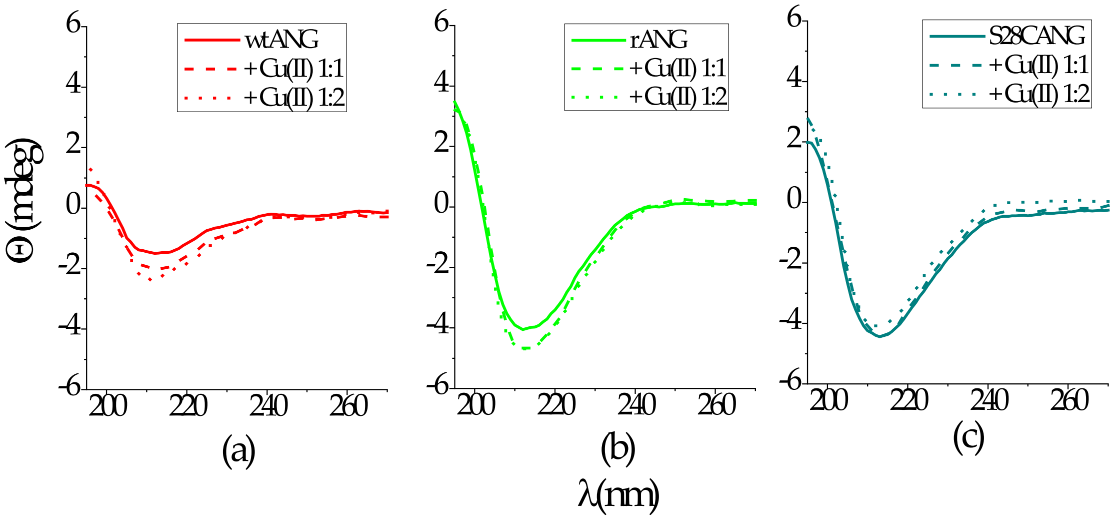

Figure S1. Far-UV CD spectra of (**a**) wtANG (red, solid-line), (**b**) rANG (green, solid-line), (**c**) S28CANG (cyan, solid-line) before and after the addition of 1 and 2 Cu(II) equivalents (dash, dot line), at the protein concentration of 2 × 10^6 M in 1 mM MOPS buffer (pH = 7.4).

MTT Assay

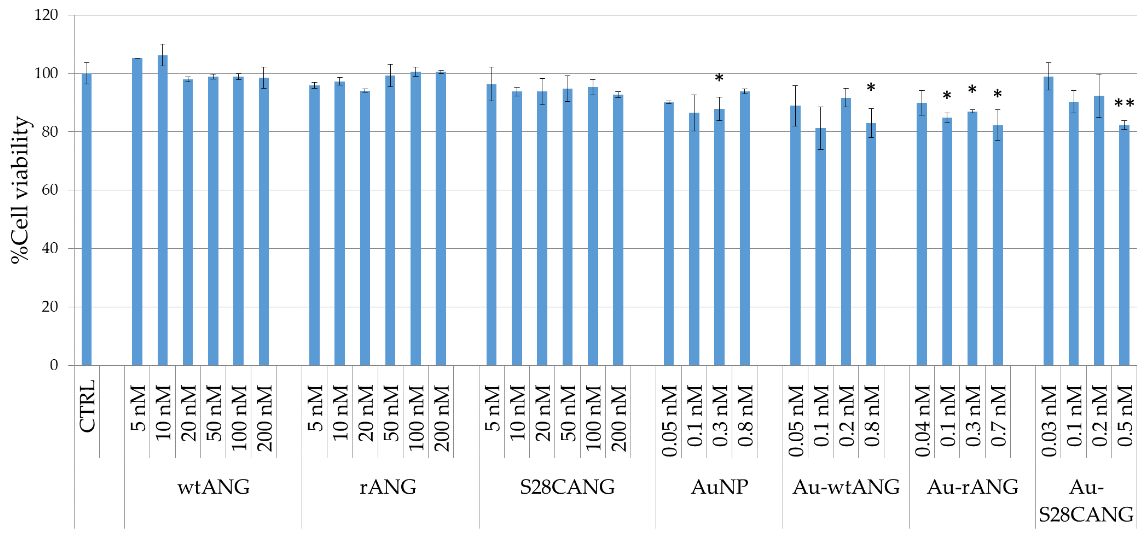

Figure S2. Cell viability assay (MTT) on HUVECs treated for 24 h with: free ANG proteins (concentration range from 5×10^{-9} M to 2×10^{-7} M); bare AuNP (concentration range from 5×10^{-11} M (= 2.2×10^{6} NP/mL) to 8×10^{-10} M (= 3.5×10^{7} NP/mL)); AuNP-ANG hybrids (Au-wtANG: concentration range from 5×10^{-11} M (= 4.8×10^{5} NP/mL) to 8×10^{-10} M (= 8.2×10^{6} NP/mL); Au-rANG: concentration range from 4×10^{-11} M (= 2.8×10^{5} NP/mL) to 7×10^{-10} M (= 4.6×10^{6} NP/mL); Au-S28CANG: from 3×10^{-11} M (= 1.5×10^{5} NP/mL) to 5×10^{-10} M (= 2.4×10^{6} NP/mL)). Statistical analysis was performed by pairwise Student's T-test. (*) $p < 0.05$, (**) $p < 0.01$ vs. CTRL. The bars represent means ± S.D. of three independent experiments performed in triplicate (S.D. = standard deviation).

Endothelial Cells Treatment

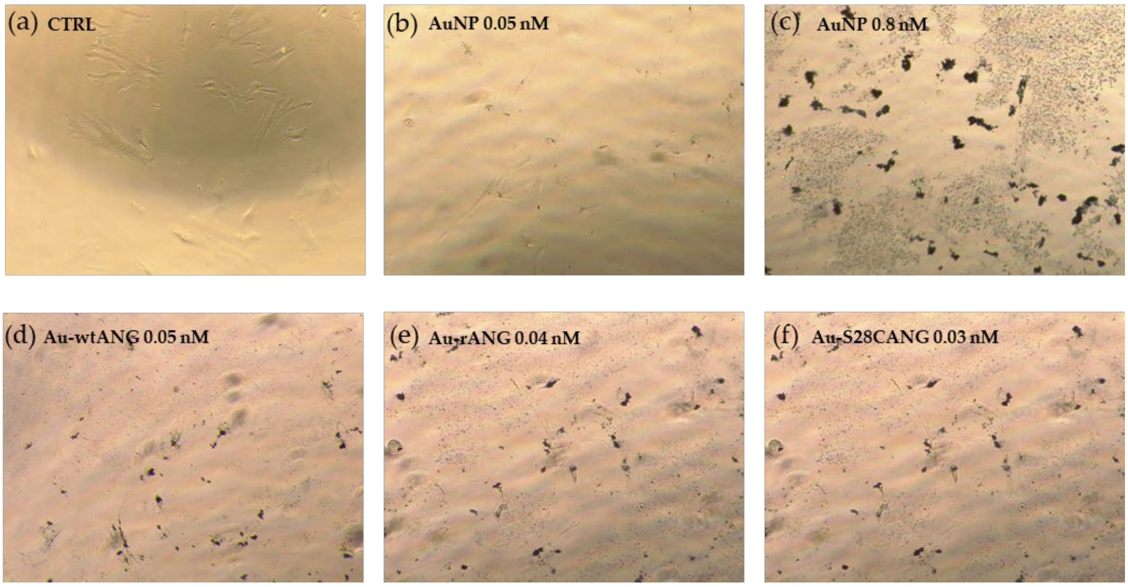

Figure S3. Optical bright field micrographs of endothelial cells (HUVEC) untreated (**a**) and after 24 h treatment with (**b**) AuNP 0.05 nM (2.2×10^{6} NP/mL), (**c**) AuNP 0.8 nM (3.5×10^{7} NP/mL), (**d**) Au-wtANG 0.05 nM (4.8×10^{5} NP/mL), (**e**) Au-rANG 0.04 nM (2.8×10^{5} NP/mL), (**f**) Au-S28CANG 0.03 nM (1.5×10^{5} NP/mL). The black dots are AuNP aggregated inside the cells.
